# A facultative endosymbiont in aphids can provide diverse ecological benefits

**DOI:** 10.1111/jeb.12705

**Published:** 2015-08-18

**Authors:** E. R. Heyworth, J. Ferrari

**Affiliations:** ^1^ Department of BiologyUniversity of YorkYorkUK

**Keywords:** *Acyrthosiphon pisum*, cost, facultative endosymbiont, multiple ecological effects, X‐type (PAXS)

## Abstract

Ecologically important traits of insects are often affected by facultative bacterial endosymbionts. This is best studied in the pea aphid *Acyrthosiphon pisum*, which is frequently infected by one or more of eight facultative symbiont species. Many of these symbiont species have been shown to provide one ecological benefit, but we have little understanding of the range of effects that a single strain can have. Here, we describe the phenotypes conferred by three strains of the recently discovered bacterium known as X‐type (Enterobacteriaceae), each in their original aphid genotype which also carries a *Spiroplasma* symbiont. All comparisons are made between aphids that are coinfected with *Spiroplasma* and X‐type and aphids of the same genotype that harbour only *Spiroplasma*. We show that in all cases, infection with X‐type protects aphids from the lethal fungal pathogen *Pandora neoaphidis*, and in two cases, resistance to the parasitoid *Aphidius ervi* also increases. X‐type can additionally affect aphid stress responses – the presence of X‐type increased reproduction after the aphids were heat‐stressed. Two of the three strains of X‐type are able to provide all of these benefits. Under benign conditions, the aphids tended to suffer from reduced fecundity when harbouring X‐type, a mechanism that might maintain intermediate frequencies in field populations. These findings highlight that a single strain of a facultative endosymbiont has the potential to provide diverse benefits to its aphid host.

## Introduction

Symbiosis between animals and microbes is remarkably common, and in the last decade, it has emerged that most insect species are infected by bacterial endosymbionts (Hilgenboecker *et al*., 2008; Duron & Hurst, 2013). For the majority of species, the effect on the host is unknown, but it is clear that the symbionts can be divided into three overlapping functional groups (Moran *et al*., 2008). Primary or obligate symbionts tend to occur in insects feeding on unbalanced diets and supply the hosts with nutrients that are deficient in the diet (Douglas, 1998). Facultative symbionts can be divided into two groups: reproductive manipulators that affect the hosts' reproduction to maximize their own transmission and mutualists that can affect a wide range of life‐history and ecologically important traits (Oliver *et al*., 2010; Feldhaar, 2011; Ferrari & Vavre, 2011).

The symbionts are typically transmitted from mother to offspring, although horizontal transfer occurs at lower frequencies (Moran & Dunbar, 2006; Gehrer & Vorburger, 2012; Ahmed *et al*., 2013; Henry *et al*., 2013). The predominantly vertical transmission suggests that host and symbiont share their fate and mutualistic relationships are likely to evolve (Lively *et al*., 2005). Indeed, facultative symbionts confer a wide range of benefits to their hosts, for example protection from natural enemies (Oliver *et al*., 2003; Scarborough *et al*., 2005; Teixeira *et al*., 2008; Xie *et al*., 2010), protection from extreme temperatures (Montllor *et al*., 2002; Neelakanta *et al*., 2010) and the ability to use a greater diversity of resources (Tsuchida *et al*., 2004; Hansen & Moran, 2014). In some cases, horizontal transfer of the symbionts between insects can lead to the instant acquisition of a beneficial ecological trait (Tsuchida *et al*., 2011; Kikuchi *et al*., 2012), and the symbiont may rapidly spread through the population (Ferrari & Vavre, 2011; Himler *et al*., 2011; Cockburn *et al*., 2013).

Aphids and specifically the pea aphid, *Acyrthosiphon pisum*, are among the best studied insect–symbiont systems. The pea aphid alone is known to host at least eight different facultative symbiont species (Sandström *et al*., 2001; Tsuchida *et al*., 2010; Russell *et al*., 2013), with typically zero to four species within the same aphid individual (Ferrari *et al*., 2012; Henry *et al*., 2013; Russell *et al*., 2013). Several of these species are well known for one particular ecological effect. For example, *Hamiltonella defensa* carries a lysogenic bacteriophage that protects the aphid from the parasitoid wasp *Aphidius ervi* (Oliver *et al*., 2003, 2009; Degnan & Moran, 2008), whereas *Regiella insecticola* is known for providing resistance to a fungal pathogen (Scarborough *et al*., 2005), a benefit that other species of endosymbionts, including *Spiroplasma*, have also been shown to confer (Łukasik *et al*., 2013b).

However, at present, little is known about the breadth of ecological effects that a single strain of symbiont can have, predominantly because experiments on different traits tend to be performed by different research groups. Exceptions are a strain of *H. defensa* and a strain of *Serratia symbiotica* that can both improve resistance to a parasitoid and increase tolerance to heat shock albeit to different degrees (Oliver *et al*., 2003; Russell & Moran, 2006). Understanding the diversity of effects that a single genotype can have is important for explaining the distribution of symbionts in insect populations. A possible explanation for the typically intermediate frequencies is that different symbionts provide different benefits and are therefore selected for in divergent ecological scenarios (Oliver *et al*., 2014). To test this hypothesis, a more complete characterization of single host–symbiont combinations, as well as of the variation within symbiont species, is needed.

An alternative explanation for variable frequencies is that carrying a symbiont comes at a cost. Evidence for such costs is extremely variable between studies, with some documenting no costs under benign conditions and others a reduction in fitness of about 10–20% compared with uninfected hosts (Sakurai *et al*., 2005; Simon *et al*., 2007; Chandler *et al*., 2008). In particular, many but not all *H. defensa* strains cause a reduction in longevity and therefore also lifetime fecundity (Simon *et al*., 2011; Vorburger & Gouskov, 2011; Tsuchida *et al*., 2013).

Here, we employ the recently discovered symbiont known as X‐type or PAXS (Pea Aphid X‐type symbiont) (Enterobacteriaceae) (Guay *et al*., 2009) and provide a representative overview of its effects on the aphid phenotype, namely resistance to two natural enemies from two different kingdoms, resistance to heat shock and fitness under benign conditions. X‐type is found commonly in field populations, with prevalences of up to 45% (Ferrari *et al*., 2012; Russell *et al*., 2013), and has also been found in other aphid species (Lamelas *et al*., 2008; Henry *et al*., 2015). The first study published on its effects correlates natural double infections of X‐type and *H. defensa* with a high level of physiological defence against the parasitoid *Aphidius ervi* under heat stress (Guay *et al*., 2009).

The aphid genotypes used in this study were all naturally coinfected with X‐type and a *Spiroplasma* symbiont. Coinfections of multiple symbionts are common in aphids (Ferrari *et al*., 2012; Smith *et al*., 2015), and the presence of one species has the potential to alter the other species' effect on the host's phenotype (Oliver *et al*., 2006). For example, it is possible that carrying two symbiont species increases the costs imposed on the host (Oliver *et al*., 2006).

Here, we used three pea aphid genotypes that are naturally infected with X‐type and *Spiroplasma*, and cured them of X‐type, giving us six aphid lines in total. This allowed comparison of X‐type‐mediated effects within each of the aphid genotypes. We tested the following hypotheses: (i) X‐type can provide multiple ecological benefits to the host (resistance to heat shock, a parasitoid and a lethal fungal pathogen). (ii) There is genetic variation in the degree to which these benefits are conferred by X‐type, which is due to variation between X‐type strains or an interaction between X‐type and the genotype of the host or other symbionts. (iii) Carrying X‐type is costly for the aphid under benign conditions.

## Materials and methods

### Aphids

The pea aphid [*Acyrthosiphon pisum* (Harris)] reproduces parthenogenetically throughout spring and summer, allowing genetically identical individuals to be maintained indefinitely in the laboratory. Pea aphids feed on a variety of different legume species, but individuals are highly specialized on single plant species (Via, 1991; Ferrari *et al*., 2008) and populations found on different plants are genetically differentiated (Peccoud *et al*., 2009; Ferrari *et al*., 2012). Despite this specialization, almost all pea aphids perform well on broad bean, *Vicia faba* (L.) (Ferrari *et al*., 2008). For this study, three pea aphid clonal genotypes were used (codes 217, 322, 324), each collected from the UK and naturally infected with X‐type and *Spiroplasma*, a bacterial species that cannot yet be cured and so was a consistent background across all lines. The aphids were also screened for *H. defensa*,* R. insecticola, S. symbiotica*,* Rickettsia* sp. and *Rickettsiella viridis* using the diagnostic PCR protocols described in Ferrari *et al*. (2012) and in Tsuchida *et al*. (2010) for *R. viridis*; none of these symbionts were detected.

The aphids were cured from the X‐type infection by feeding on leaves placed in an antibiotic cocktail of 1% Ampicillin, 0.5% Gentamicin and 0.5% Cefotaxime (McLean *et al*., 2011), leading to a total of six aphid lines. Aphids were left for at least six months after curing and retested for endosymbionts regularly to ensure that both the natural infection and the cured lines were stably maintained. To check for contamination between lines, the aphid genotype was regularly confirmed by screening four microsatellite loci (Ferrari *et al*., 2008).

Our choice of aphid lines requires some caution in the interpretation of our results. Each pair of uninfected and infected lines consisted of a different aphid genotype and potentially a different genotype of the primary symbiont *Buchnera aphidicola* or the *Spiroplasma* symbiont (for simplicity, we will refer to these as ‘aphid genotype’ in the results). Any variation that we observe between the three pairs in the phenotypic effects of X‐type may therefore be due to genetic variation between X‐type strains or a genotypic interaction between X‐type and the other three species. Based on the sequences of six household genes, there is little genetic variation between strains of X‐type and no variation has been found for the three strains used here (Henry *et al*., 2013).

Genotypes 322 and 324 were collected in 2008 from *Trifolium pratense* L. and genotype 217 in 2010 from *Medicago sativa* L., all in the South of England. Unless otherwise noted, experiments were performed at 20 °C and long‐daylight conditions of 16‐h:8‐h light:dark with a relative humidity of 40 ± 15%. Aphids were reared on *Vicia faba* cv. ‘The Sutton’ leaves or seedlings. Aphids were kept in simultaneously refreshed cultures and to reduce maternal effects were raised in smaller groups prior to use in experiments.

### Performance under benign conditions and after heat shock

Fecundity is a basic measure of fitness, and we tested the difference between cured lines and those infected with X‐type under benign conditions and after exposure to heat shock. Heat stress can render aphids essentially incapable of reproduction (Montllor *et al*., 2002), but some facultative endosymbionts have been shown to maintain aphid survival and reproduction after heat shock (Montllor *et al*., 2002; Russell & Moran, 2006).

For each line, groups of ten two‐day‐old aphids that had been reared at 20 **°**C were put in cages formed by placing a plastic, vented 2 L cage over a pot containing four *V. faba* seedlings. The replicates were then divided into two treatment groups, one group to be tested at 20 **°**C and the other exposed to heat shock. In the heat‐shock treatment, the temperature rose consistently from 20 **°**C to 38.5 **°**C over a period of two hours; this temperature was maintained for four hours and then decreased to 20 **°**C over another two hours. All plants, including those of the controls, were exchanged on the day after the heat shock to ensure consistent plant quality. The proportion of surviving aphids was counted seven days after the heat shock. One surviving apterous aphid per pot was put on a petri dish containing a broad bean leaf and its number of offspring recorded daily for eighteen days, at which point the vast majority of aphids were dead or had stopped reproducing. For survival measures, there were seven to nine replicates for each aphid line; for fecundity, there were between five and nine (mean 6.9) across two temporal blocks.

### Performance on the original collection plant species

In addition to measuring fecundity on *V. faba*, we looked at whether infection with X‐type affects the reproduction and survival on the plant that the aphids were specialized on (assumed to be the species that the aphids were collected from). Young adult aphids were put on Petri dishes containing leaves from the plant species that the aphids were originally found on (*Trifolium pratense* for 322 and 324*, Medicago sativa* for 217) placed in 2% agar. On the following day, their offspring were used to create groups of five one‐day‐old aphids on Petri dishes containing the same plant species. Nine days later, the number surviving from each group was counted. From each group, one apterous individual was placed on its own *T. pratense* or *M. sativa* plate and offspring counted daily for eighteen days. The dishes were exchanged regularly. The number of replicates was six per aphid line for survival and four to five for fecundity.

Genotype 217 performed very poorly on *M. sativa*, suggesting that it might have been a migrant or hybrid specialized on a different plant species. It was collected from a field where both *M. sativa* and *T. pratense* were grown and we therefore repeated the experiment with 217 on *T. pratense* as well as *M. sativa*. We pooled the *M. sativa* data for both experiments.

### Resistance to the parasitoid *Aphidius ervi*

Parasitoid wasps are a major source of aphid mortality (Müller & Godfray, 1998; Schmidt *et al*., 2003). The wasps oviposit into aphid nymphs and their larvae develop inside the living aphid. The larvae pupate after approximately one week, killing the aphid. The aphid then forms the so‐called mummy, out of which the adult parasitoid eventually emerges. *Aphidius ervi* Haliday (Hymenoptera, Braconidae) attacks a range of aphid species and is commonly found on pea aphids. To investigate whether X‐type affects the parasitoid resistance of its hosts, aphids were exposed to *A. ervi* females (Ferrari *et al*., 2001). Thirty four‐day‐old aphids from each aphid line were placed on *V. faba* plants enclosed in a clear plastic, vented cage. A single *A. ervi* female that had emerged up to 24 h earlier was added to each cage for nine hours to forage for aphids and to oviposit. After ten days, mummies had formed from aphids that had been successfully parasitized and were counted along with any surviving aphids. This type of parasitism assay is routinely used to assess physiological resistance to parasitoids in aphids (e.g. Henter & Via, 1995; Ferrari *et al*., 2001; Oliver *et al*., 2003). It is possible that there are differences in parasitoid oviposition behaviour between aphid lines, but typically this found in choice rather than no‐choice situations such as the assay used here (Henter & Via, 1995; Oliver *et al*., 2003, 2012; Łukasik *et al*., 2013a). The total number of replicates for each aphid line varied between four and ten (with a mean of 6.8), spread between two temporal blocks. The blocks were pooled for the statistical analysis as no significant difference was found between blocks.

### Resistance to the fungal pathogen *Pandora neoaphidis*

Under warm and humid conditions, entomopathogenic fungi are a further major cause of aphid mortality. These fungi grow in the aphid's body and, like parasitoids, kill the host after a few days. We assessed aphid resistance to the pathogenic fungus *Pandora neoaphidis* (Remaudière & Hennebert) Humber (Zygomycetes; Entomophorales) (isolate reference X4, Rothamsted Research collection). To produce the experimental aphids, young adult aphids were placed on Petri dishes with *Vicia faba* leaves and left overnight before being removed. Once the offspring were ten days old, they were assembled in groups of twenty. Each group was exposed to a pair of adult fungus‐killed aphids that had been placed on damp filter paper suspended from the lid of a Petri dish and been left overnight at 20 °C and high humidity to start the fungus sporulating. These aphid cadavers created a spore shower over a small plastic tube (height 50 mm, diameter 15 mm) that contained the group of 20 aphids. After 90 min, the sporulating cadavers were removed and the populations placed on fresh two‐week‐old *Vicia faba* plants. For the next two weeks, plants were checked every few days and sporulating aphids removed and counted.

As all six aphid lines are infected with *Spiroplasma*, which can confer resistance to *P. neoaphidis* (Łukasik *et al*., 2013b), we included a fourth genotype, known as 145. This genotype does not carry any known facultative symbionts and is susceptible to this strain of *P. neoaphidis*. It was included as a control to ensure the protocol worked, in case infection with *Spiroplasma* made the target aphids fully resistant.

### Statistical analysis

Data were analysed using the r package 3.2.1 (R Core Team, 2012). Survival data were analysed using analysis of deviance, assuming a quasibinomial error distribution. All other data (except susceptibility to the fungal pathogen) were subjected to an analysis of variance and the data were transformed when necessary to meet model assumptions (square‐root transformation for fecundity, arcsine‐square‐root transformation for susceptibility to the parasitoid). Aphid genotype, presence of X‐type and their interaction were the explanatory variables plus heat‐shock treatment and its interactions in the heat‐shock experiment. The model assumptions were checked with Shapiro–Wilk normality tests and Levene's test for homogeneity of variances. When an explanatory variable with more than two levels or an interaction was significant, we performed post hoc tests using Holm's correction for multiple testing in the package ‘phia’ in the r software. The data for fungus susceptibility could not be transformed to meet anova assumptions and were therefore analysed using nonparametric tests (Mann–Whitney *U*‐test for the presence of X‐type and Kruskal–Wallis test for aphid genotype).

## Results

### Performance under benign conditions and after heat shock

As expected, the heat‐stressed insects had significantly lower seven‐day survival than the control aphids (*F*_1, 87 _= 40.39, *P *<* *0.001), showing that the heat stress had a negative effect on the aphids. Survival was not significantly affected by any other factor, including the presence of X‐type (*F*_1, 87 _= 0.20, *P *=* *0.65), aphid genotype (*F*_2, 87 _= 1.07, *P *=* *0.35) or their interaction (*F*_2, 87 _= 2.17, *P *=* *0.12).

**Figure 1:**
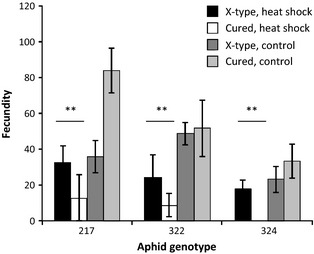
The effect of infection with X‐type on the fecundity of three pea aphid genotypes feeding on *Vicia faba*. The figure shows a comparison between aphids that are naturally infected with X‐type and *Spiroplasma* (black and dark grey bars) or cured from X‐type, but still infected with *Spiroplasma* (white and light grey bars). The black and white bars show fecundity after a heat‐shock treatment; dark and light grey bars show fecundity at 20 °C. Means and standard errors are shown. The asterisks indicate the results of post hoc tests: there is a significant difference between aphids infected with X‐type and cured aphids in the heat‐shock treatment, but not under control conditions (**P <* *0.01).

Heat shock also reduced the fecundity of the aphids (Fig. 1; *F*_1, 68 _= 47.65, *P *<* *0.001) and the three aphid genotypes differed intrinsically in their fecundity irrespective of symbiont infection (*F*_2, 68 _= 3.43, *P *=* *0.04). There was no overall effect of infection with X‐type on fecundity (*F*_1, 68 _= 1.34, *P *=* *0.25), but it differed between treatments (heat shock × infection with X‐type: *F*_1, 68 _= 12.19, *P *<* *0.001): aphids infected with X‐type had higher fecundity after heat shock but tended to have lower numbers of offspring in the control treatment. This effect did not differ significantly between the three aphid genotypes (heat shock × infection with X‐type × aphid genotype: *F*_2, 68 _= 2.37, *P *=* *0.10), even though Fig. 1 suggests that a cost of carrying X‐type under benign conditions occurred in genotype 217.

### Performance on the original collection plant species

The aphids' performance was tested on the *Trifolium pratense*. Genotype 217 was additionally tested on *M. sativa*, see methods. On *T. pratense*, survival was high with little variation, but there was a small effect of aphid genotype with genotype 322 having poorer survival overall (Fig. S1; *F*_2, 28_ = 4.49, *P *=* *0.02). There was neither a significant effect of X‐type on survival (*F*_1, 28_ = 3.13, *P *=* *0.09) nor an interaction of X‐type with aphid genotype (*F*_2, 28 _= 0.36, *P *=* *0.70). For genotype 217, the presence of X‐type also had no effect on survival on *M. sativa* (Fig. S2a; *F*_1, 31_ = 1.81, *P *=* *0.19).

**Figure 2:**
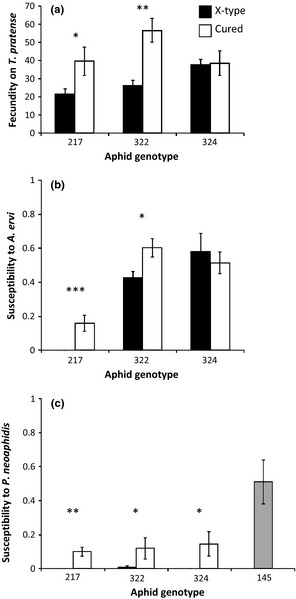
The effect of infection with X‐type on three pea aphid genotypes. (a) Fecundity on *Trifolium pratense*. (b) Susceptibility to the parasitoid *Aphidius ervi*. (c) Susceptibility to the fungal pathogen *Pandora neoaphidis*, which includes a control aphid genotype that is known to be susceptible and not infected with facultative symbionts (genotype 145, grey bar). All panels show a comparison between aphids that are naturally infected with X‐type and *Spiroplasma* (black bars) or cured from X‐type, but still infected with *Spiroplasma* (white bars). Means and standard errors are shown. The asterisks denote significant differences between lines infected with X‐type and cured from X‐type within aphid genotypes (*P <* *0.05, **P <* *0.01, ***P <* *0.001).

The fecundity on *T. pratense* did not differ between aphid genotypes (Fig. 2a; *F*_2, 24_ = 1.90, *P *=* *0.17). It was overall reduced by the presence of X‐type (*F*_1, 24_ = 11.37, *P *=* *0.003), a cost that did not occur in genotype 324 (aphid genotype × infection with X‐type: *F*_2, 24_ = 3.42, *P *=* *0.049). The presence of X‐type also reduced the fecundity of genotype 217 on *M. sativa* (Fig. S2b; *F*_1, 27_ = 9.91, *P *=* *0.004).

### Resistance to the parasitoid *Aphidius ervi*

We tested whether X‐type provides resistance to the parasitoid *A. ervi*. Overall, X‐type caused a significant decrease in susceptibility to the parasitoid (Fig. 2b; *F*_1, 34 _= 11.61, *P *=* *0.002), and there was a clear difference between aphid genotypes (*F*_2, 34 _= 61.95, *P *<* *0.001) with genotype 217 being overall highly resistant. There was a significant interaction between genotype and infection status (*F*_2, 34 _= 5.02, *P *=* *0.01) as X‐type had no effect on resistance in genotype 324.

### Resistance to the fungal pathogen *Pandora neoaphidis*

Aphid genotype 145 was highly susceptible to *P. neoaphidis*, showing that the spore shower protocol worked (Fig. 2c). The other aphid genotypes were more resistant to the pathogen, suggesting that *Spiroplasma* may be providing a degree of resistance to these aphids. Aphids infected with X‐type were significantly more resistant to the fungus (*W *=* *268.5, *P *<* *0.001), which was also found in separate tests on each aphid genotype (217: *W *=* *38.5, *P *<* *0.01; 322: *W *=* *25.5, *P *<* *0.05; 324: *W *=* *24.0, *P *=* *0.05). There were no significant differences between the three aphid genotypes (Kruskal–Wallis *Χ* ^2^_2_ = 0.24, *P *=* *0.89).

## Discussion

We found strong evidence that infection with the endosymbiont X‐type can provide multiple and ecologically diverse benefits, which include tolerance to heat shock and resistance to natural enemies from two different kingdoms. However, whereas some strains of X‐type appear to be able to confer all of these benefits, others provide only a subset. Harbouring X‐type under benign conditions can carry a cost in terms of aphid fecundity, especially on the original host plant species.

All of the benefits observed in this study have previously been found for other insect–endosymbiont combinations (e.g. Montllor *et al*., 2002; Oliver *et al*., 2003; Scarborough *et al*., 2005), but to our knowledge, no single insect–symbiont genotype combination has been tested for all of these (or a similar range of other benefits) and only one strain of *H. defensa* and one strain of *S. symbiotica* have been shown to provide more than one benefit (Oliver *et al*., 2003; Russell & Moran, 2006). A possible explanation for the diversity of endosymbiont species in insect populations is that separate species provide different benefits and that the host is selected for carrying the symbiont most suited to its environmental conditions (Oliver *et al*., 2014). Given that our study is one of the very few examples where a single strain has been tested for multiple benefits, our results suggest that it may be relatively common that a symbiont provides multiple benefits and therefore it might be adaptive to carry the same symbiont in a wide range of conditions.

We have also shown that not all X‐type strains employed here show all of the benefits and there is functional variation between the three combinations of genotypes. These differences between X‐type strains are unexpected because there are no differences in six household genes of the three X‐type strains (Henry *et al*., 2013) and two of the aphid genotypes (322 and 324) are closely related and were collected in close proximity, but the simplest explanation of this result is that there is variation in functional genes between the three X‐type strains. However, caution must be taken when interpreting this result, because each X‐type strain was tested in its original host genotype, with additional potential genetic variation between the primary symbiont *Buchnera* and the *Spiroplasma* strains present. It is therefore possible that the observed strain variation is actually due to an interaction between X‐type genotype and the genotype of at least one of the other three players in the system, and future experiments are needed to address this directly. Whether the observed variation is due to interactions between genotypes or not has implications for the dynamics of the system, in particular if the symbiont is horizontally acquired by a different aphid genotype (Russell *et al*., 2003).

Similarly, the presence of *Spiroplasma* in both the original lines and those cured of X‐type complicates the interpretation. The most straightforward explanation of the observed patterns is that X‐type alters the host's phenotype directly without interacting with *Spiroplasma*. It is possible that X‐type is merely enhancing an effect that is actually caused by *Spiroplasma* rather than providing direct benefits, or other interactions between the symbionts are impacting the host. For example, *Spiroplasma*'s only known benefit, providing resistance to the fungal pathogen *P. neoaphidis* (Łukasik *et al*., 2013b), may be increased by the presence of X‐type. Experiments with single infections of X‐type are required to clarify this issue, but unfortunately we were unable to find such natural lines before performing our experiments. However, the difference between the cured lines and their counterparts infected with X‐type will be caused by the presence of X‐type, whether this is a direct or indirect effect.

The presence of X‐type used here exerted a fecundity cost on their host, with a reduction in fecundity of up to 50% on the natural host plant species *T. pratense*. Costs on this magnitude have previously only been reported for artificial host–symbiont associations (Cayetano & Vorburger, 2013; Tsuchida *et al*., 2013) which may be because of incompatibilities between particular genotypes. Natural infections usually appear to come at a lower cost (Sakurai *et al*., 2005; Simon *et al*., 2007; Chandler *et al*., 2008) or a cost that can only be detected under more stressful conditions (Oliver *et al*., 2006; Dykstra *et al*., 2014). These high costs are likely to outweigh some of the benefits provided by X‐type, and if these are representative of costs suffered under field conditions, it is surprising that X‐type is maintained at relatively high frequencies in aphid populations. It is also possible that the high cost is a result of the coinfection with *Spiroplasma* as coinfections may require more resources from the host than single infections (Oliver *et al*., 2006).

In conclusion, we have shown that a single endosymbiont can confer a wide range of ecological benefits to its host at a considerable cost. This study further highlights the variability between strains or genotypic interactions with the host or other symbionts demonstrating the difficulty of predicting the dynamics in natural populations.

## Supporting information


**Figure S1** The effect of infection with X‐type on the survival of three pea aphid genotypes feeding on *Trifolium pratense*.
**Figure S2** The effect of infection with X‐type on (a) survival and (b) fecundity in pea aphid genotype 217 on when feeding on *Medicago sativa*.
