# Supplementary material for: A facultative endosymbiont in aphids can provide diverse ecological benefits
Source: J Evol Biol. 2015 Aug 18;28(10):1753–60. doi: 10.1111/jeb.12705 (PMC4949989; doi:10.1111/jeb.12705)
Supplement: Supplementary file 1 — Figure S1 The effect of infection with X‐type on the survival of three pea aphid genotypes feeding on Trifolium pratense. Figure S2 The effect of infection with X‐type on (a) survival and (b) fecundity in pea aphid genotype 217 on when feeding on Medicago sativa. [file JEB-28-1753-s001.docx]

A facultative endosymbiont in aphids can provide diverse ecological benefits

**Eleanor R. Heyworth and Julia Ferrari^1^**

Department of Biology, University of York, York, YO10 5DD, United Kingdom

## Supplementary Figures

**
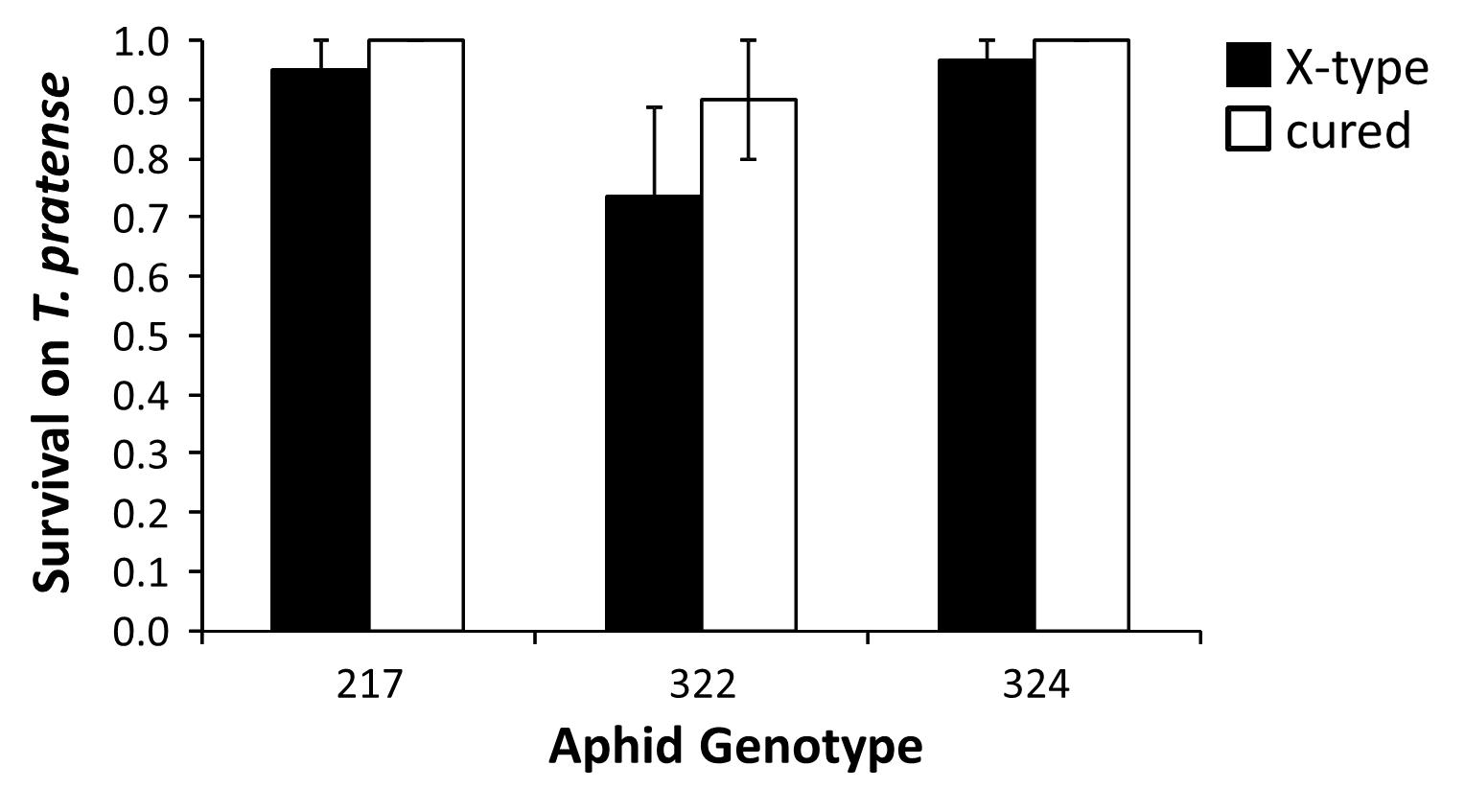
**

**Figure S1.** The effect of infection with X-type on the survival of three pea aphid genotypes feeding on *Trifolium pratense*. The figure shows a comparison between aphids that are naturally infected with X-type and *Spiroplasma* (black bars) or cured from X-type, but still infected with *Spiroplasma* (white bars). Means and standard errors are shown.


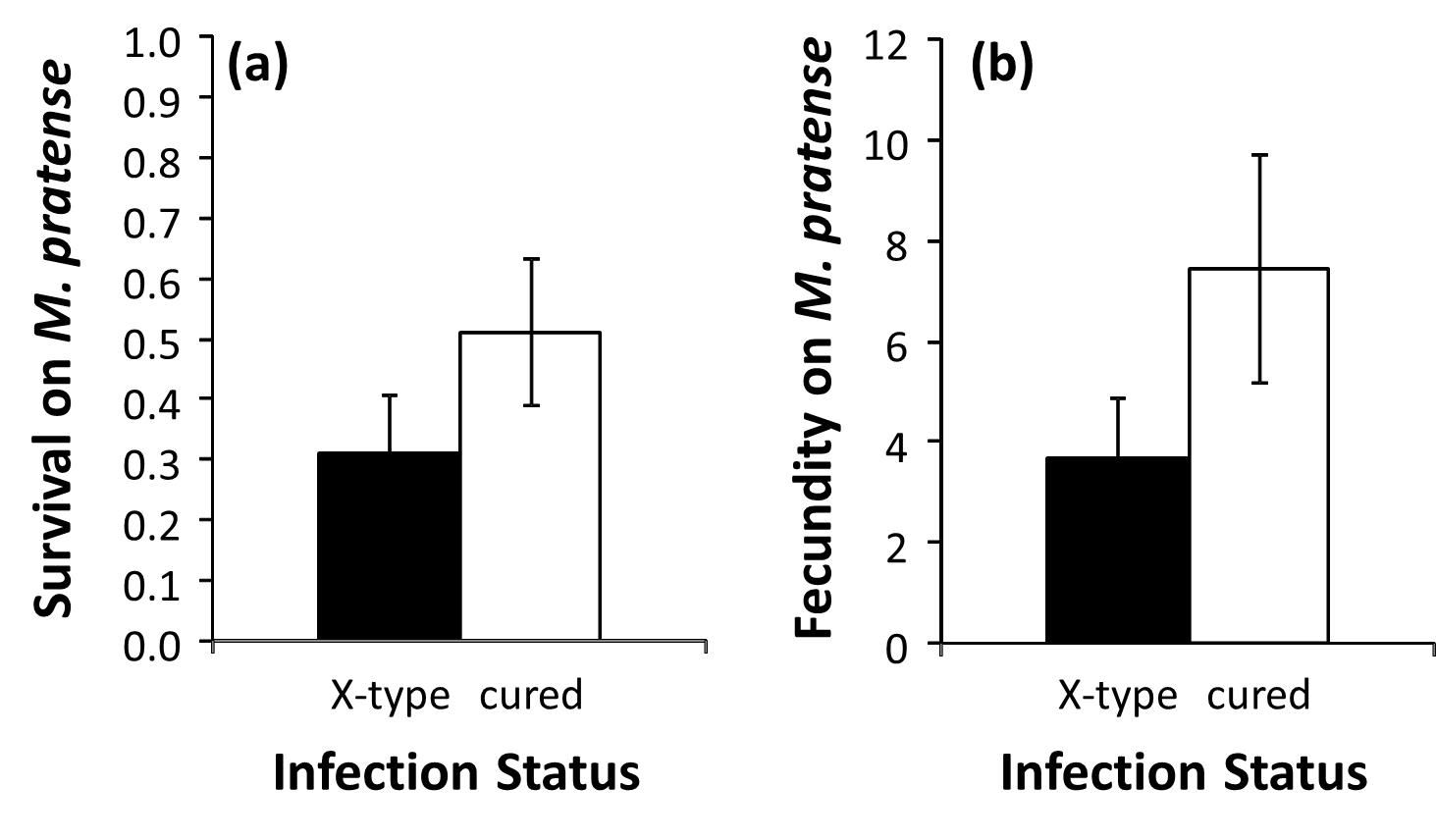


**Figure S2.** The effect of infection with X-type on (a) survival and (b) fecundity in pea aphid genotype 217 on when feeding on *Medicago sativa*. The figure shows a comparison between aphids that are naturally infected with X-type and *Spiroplasma* (black bars) or cured from X-type, but still infected with *Spiroplasma* (white bars). Means and standard errors are shown.
